# Real-world chest radiograph findings and clinical consequences in preschool children with recurrent wheezing

**DOI:** 10.1186/s12887-026-07321-4

**Published:** 2026-07-15

**Authors:** Emma Lück, Alisa Jovanović, Alina-Marielle Ockelmann, Annelie Pauline Reihle, Jan Christoph Thomassen, Robert Walter Körner

**Affiliations:** Department of Pediatrics, Faculty of Medicine and University Hospital Cologne, University of Cologne, Kerpener Str. 62, Cologne, 50937 Germany

**Keywords:** Recurrent wheezing, Preschool children, Episodic wheezing, Chest radiography, Diagnostic imaging

## Abstract

**Purpose:**

Recurrent wheezing in young children often prompts chest X-rays (CXRs) during diagnostic evaluation, despite limited evidence supporting their clinical utility. This study aimed to assess whether CXRs provide diagnostic or therapeutic benefit in children under five years of age with recurrent wheezing.

**Methods:**

We conducted a retrospective review of 271 children under five years of age who presented with recurrent wheezing to a pediatric pulmonology outpatient clinic at the University Hospital of Cologne, Germany, between 2010 and 2022. All included children underwent CXR as part of their diagnostic workup. Data on clinical characteristics, CXR findings, and downstream clinical decisions were analyzed.

**Results:**

Further diagnostic workup was initiated in only 1% of cases, and no changes in diagnosis or treatment were documented. Despite these limited clinical consequences, abnormal CXR findings were reported in 73% of cases. The most frequent abnormality was peribronchial cuffing (35%). Most clinical parameters—including age, sex, prematurity, and atopic conditions—were not associated with abnormal CXR findings and therefore did not identify a subgroup of children who might benefit from selective imaging. Elevated blood eosinophil counts (≥ 150/µL, *p* = 0.023) were the only factor associated with abnormal CXR findings.

**Conclusion:**

In this real-world cohort of preschool children with recurrent wheezing, abnormal chest radiograph findings were frequent but largely nonspecific and rarely led to changes in diagnosis or clinical management. The radiographic patterns observed suggest the presence of underlying lower airway inflammation even outside of acute episodes. These findings add empirical evidence that is consistent with current recommendations advocating a clinically driven rather than routine use of chest radiography in preschool children with recurrent wheezing.

## What is known

- Recurrent wheezing is common in preschool children, and chest radiographs are frequently obtained during diagnostic evaluation.
- Current clinical guidelines do not recommend routine chest radiography in preschool children with recurrent wheezing in the absence of atypical or severe features.

## What is new

- In a large real-world cohort of preschool children with recurrent wheezing, abnormal chest radiograph findings were frequent but predominantly nonspecific and rarely influenced diagnosis or management.
- This study adds real-world evidence supporting selective rather than routine use of chest radiographs in preschool recurrent wheezing.

## Introduction

Recurrent wheezing is a highly prevalent and significant health concern in early childhood. Approximately half of all children under the age of three wheeze at least once [1]. A substantial proportion of these children experience recurrent wheezing. This condition is commonly referred to as “preschool wheezing”, encompassing children up to the age of five or six years. It represents one of the most common reasons for pediatric consultations, emergency department visits, and referrals to pediatric pulmonologists [2]. Since wheezing is a symptom-based diagnosis, it is typically made through history-taking and confirmed by physical examination [3]. The symptom burden varies widely, ranging from minimal impairment to severe respiratory distress and frequent hospitalisations [4]. Recurrent wheezing can be categorized into different phenotypes, including distinctions based on the timing of onset and symptom persistence—such as transient early, late-onset, and persistent wheezing [4]. Further, trigger-based classification differentiates episodic viral wheeze—wheezing triggered exclusively by respiratory infections, with symptom-free periods in between—from multiple-trigger wheeze, in which wheezing can also occur outside of infectious episodes and can be provoked by other factors such as physical exertion, allergens, or environmental changes [5]. However, it is now recognized that children may shift between these phenotypes over time, and that the underlying pathophysiology cannot be fully captured by clinical observation alone [6]. Recurrent wheezing likely stems from a diverse and multifactorial pathophysiology, involving both host factors (e.g., lung development, immune response) and environmental factors (e.g., pathogens, allergens, pollutants), all of which contribute to bronchial obstruction.

Despite substantial progress, important knowledge gaps remain—particularly regarding the most effective diagnostic and therapeutic strategies. Diagnostic evaluations may include blood tests, allergy assessments, bronchoscopy, or esophageal pH monitoring [7]. Although pulmonary function testing is rarely feasible in preschool children, alternative diagnostic methods such as impulse oscillometry (IOS) and fractional exhaled nitric oxide (FeNO) have recently been proposed for physiological characterization and risk stratification of early wheezing disorders [8]. Likewise, lung ultrasound (LUS) has emerged as a radiation-free imaging modality with potential diagnostic value in wheezing [9, 10]. Nevertheless, these techniques are not yet established in the routine diagnostic approach to recurrent wheezing. Consequently, chest X-rays are still frequently used in clinical practice to exclude pulmonary abnormalities, although current guidelines do not recommend CXRs as part of the diagnostic work-up [11]. Given the risks associated with radiation exposure in young children and procedural stress, it is crucial to better understand the true diagnostic value of CXRs in this population. We therefore retrospectively analyzed medical records of preschool children referred to our outpatient clinic for evaluation of recurrent wheezing. The primary objective was to determine whether chest radiograph (CXR) findings resulted in changes in diagnosis or clinical management. To address this objective, we first described the spectrum of radiographic abnormalities identified in preschool children with recurrent wheezing and assessed their clinical consequences. We then explored whether clinical characteristics and established risk factors, including atopic disease, were associated with abnormal CXR findings in order to identify potential clinical ‘red flags’ that might justify selective use of CXR.

## Materials and methods

### Study design

**Fig. 1:**
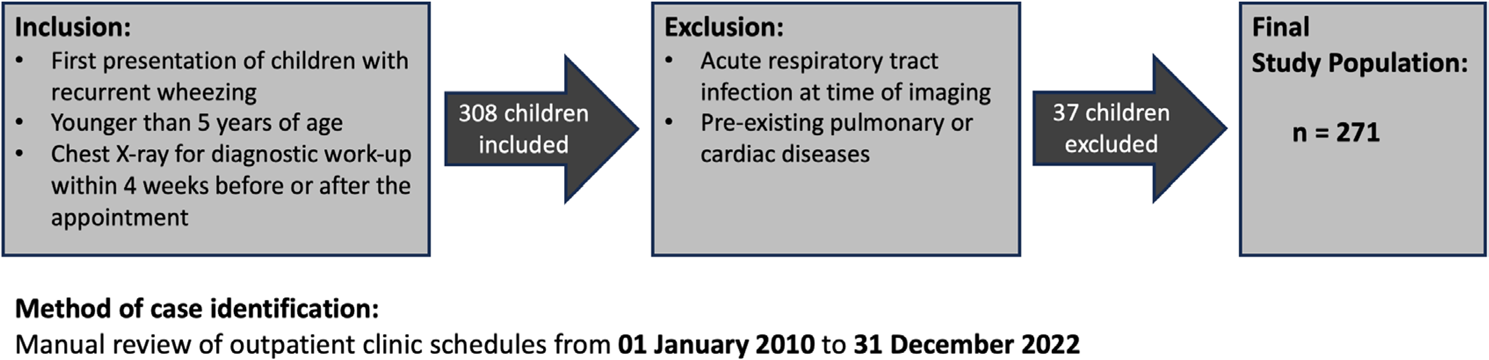
Patient selection flowchart for children under five years of age with recurrent wheezing seen between 01 January 2010 and 31 December 2022. After applying inclusion and exclusion criteria, 271 patients were included in the analysis

We conducted a consecutive retrospective chart review of children who presented with recurrent wheezing to the pediatric pulmonology outpatient clinic at the Department of Pediatrics, University Hospital of Cologne, Germany, between 01 January 2010 and 31 December 2022. Children were either referred by a community pediatrician or scheduled by caregivers. Eligible cases were identified by systematically screening the daily outpatient clinic schedules for children presenting with recurrent wheezing during this period (Fig. 1). For each patient, only the first presentation was included to avoid duplicate entries. Inclusion criteria were children under five years of age who had undergone a CXR within four weeks before or after the initial clinic visit as part of the diagnostic evaluation. Children were excluded if they had an acute respiratory tract infection at time of imaging or pre-existing pulmonary or cardiac diseases, as these may independently lead to abnormal CXR findings. While prematurity alone was not an exclusion criterion, children with a diagnosis of bronchopulmonary dysplasia were excluded due to possible long-term pulmonary alterations. The primary outcomes were the presence of abnormalities on CXR, based on the radiologic report, and whether these findings led to further diagnostic workup, revised diagnosis, or modifications in disease management.

### Data extraction and event variables

Data were extracted from anonymized electronic medical records, including clinical notes, radiologic reports, and laboratory results. The primary outcome was the clinical consequence of CXR findings, defined as whether the examination prompted further diagnostic procedures, therapeutic changes, or revision of the initial diagnosis. Secondary outcomes included characterization of the spectrum of radiographic abnormalities and exploration of clinical characteristics associated with abnormal CXR findings that might support a selective imaging strategy. Demographic variables comprised sex, age, and gestational age. Clinical history included the age of symptom onset, wheezing phenotype (viral-induced or multiple-trigger), atopic comorbidities, parental history of atopy, and other chronic conditions. Laboratory parameters included blood eosinophil counts, total serum IgE, and sensitization to food and aeroallergens. Medication history covered the use of inhaled albuterol, inhaled corticosteroids (ICS), montelukast, and inhaled long-acting beta₂-agonists. Physical examination findings were reviewed for the presence of wheezing or crackles on auscultation. The timing of CXR acquisition in relation to the clinical visit (before, during, or after) was recorded. CXRs were evaluated according to the final reports issued by board-certified pediatric radiologists at our institution. Radiographic findings were categorized descriptively based on the terminology used in these reports. To minimize interpretive bias and avoid etiologic assumptions, we did not refer to the final diagnostic interpretation in the radiologic reports but focused on the neutral descriptive terminology used within the radiologic description. The categories included peribronchial cuffing, peribronchial opacities, parenchymal consolidation, accentuated bronchial markings, pulmonary hypertransparency, and other findings. Finally, we assessed whether individual radiographic findings were associated with further diagnostic procedures, therapeutic modifications, or revision of the initial diagnosis, and documented the rationale for these clinical decisions.

### Data analysis

We performed a descriptive statistical analysis to assess frequencies. To evaluate the statistical significance between groups, Pearson’s chi-square test was applied, and Fisher’s exact test was used for smaller sample sizes. Both one-sided and two-sided *p*-values were calculated. Relative risk (RR) and binary logistic regression analysis for odds ratios (OR) were employed when appropriate to quantify associations between clinical factors and abnormal CXR findings. A *p*-value of < 0.05 was considered statistically significant in all analyses. Statistical analyses were conducted using SPSS for macOS (version 28, IBM Corp., Armonk, NY, USA).

## Results

### Patient characteristics

Diagnoses of recurrent wheezing were established through a combination of medical history and clinical evaluation. The initial assessment was typically performed by the child’s primary pediatrician, who referred the child to the outpatient clinic. This diagnosis was subsequently confirmed by the attending pediatric pulmonologist at the hospital.

A total of 271 children were included in the study (Table 1) with a mean age of 2.53 years (SD ± 1.27, range: 4 months to 4 years and 11 months). The majority were male (63%). When stratified by age, nearly one-third (28%) were between 1 and 2 years old, while the smallest proportion (15%) were aged 4–5 years. Most children were born at term (92%), and among those born prematurely, the majority were late preterm (≥ 32 weeks gestation). The mean age of wheezing onset was 13.80 months (SD ± 11.42 months). Viral wheezing was reported in 90% of cases, with episodes typically starting after upper respiratory tract infections and rapidly progressing to obstructive bronchitis. Between episodes, these children were asymptomatic. The remaining 10% of children exhibited multiple-trigger wheezing, where wheezing was also induced by factors other than infection such as physical activity, allergens, or environmental changes like temperature shifts.

**Table 1:** Characteristics of the study population. Values are presented as counts (n) with percentages (%), means ± standard deviation (SD), and ranges where appropriate. Percentages are calculated relative to the total cohort. For parameters with incomplete data, the number of available values and their percentage relative to the overall cohort are indicated. *yrs=years*,* mo=months*,* wks=weeks*,* BDL=below detection limit*

Category	Parameter	*n* (%) or Mean ± SD (Range)
Demographics	Included children	271 (100%)
	Female	100 (37%)
	Male	171 (63%)
	Age	2.53 ± 1.27 yrs (0.36—4.95)
Age distribution	0—1 yrs	47 (17%)
	1—2 yrs	77 (28%)
	2—3 yrs	49 (18%)
	3—4 yrs	58 (21%)
	4—5 yrs	40 (15%)
Gestational age	Term	248 (92%)
	32—37 wks	21 (8%)
	28—32 wks	2 (1%)
	< 28 wks	0 (0%)
Wheezing history	Onset of wheezing	13.80 ± 11.42 mo (1—48)
	Viral wheezing	243 (90%)
	Multiple-trigger	28 (10%)
Atopic and chronic diseases	Atopic dermatitis	27 (10%)
	Allergic rhinitis	23 (9%)
	Food allergy	17 (6%)
	Parental atopic disease	119 (44%)
	Other chronic diseases	13 (5%)
Atopy associated laboratory values	Eosinophils in whole blood	313.08 ± 262.17 cells/µL (0—1147)
	Eosinophils ≥ 150/µL	47 (66%) *(available values n = 71, 26%)*
	Total IgE	77.18 ± 124.86 kU/L (BDL—665 kU/L) *(available values n = 113, 42%)*
	Sensitization to aeroallergens	49 (31%) *(available values n = 160, 59%)*
	Sensitization to food allergens	20 (14%) *(available values n = 147, 54%)*
Medication	Inhaled Albuterol	180 (66%)
	Inhaled corticosteroid	147 (54%)
	Montelukast	29 (11%)
	Inhaled long-acting β_2_-agonist	7 (3%)
Auscultation at presentation	Wheezing	33 (12%)
	Crackles	15 (6%)

Atopic conditions such as atopic dermatitis, allergic rhinitis, and food allergies affected 10%, 9%, and 6% of the cohort, respectively. Notably, 44% of the children had at least one parent with a history of atopy. Other chronic conditions were present in 5% of cases. Eosinophil counts were available for almost one-quarter of the cohort, with a mean count of 313.08/µL (SD ± 262.17/µL). Elevated eosinophil counts (≥ 150/µL) were found in 66% of cases, suggesting eosinophilic inflammation. Total serum IgE was measured in 42% of children, with a mean concentration of 77.18 kU/L (SD ± 124.86 kU/L). Specific IgE values were available in over 50% of all cases. Sensitizations to aeroallergens and food allergens were detected in 31% and 14% of tested children, respectively.

More than half of the children were receiving inhaled albuterol (66%) or ICS (54%) at the time of presentation. Montelukast was prescribed in 11% of cases, while inhaled long-acting beta₂-agonists were rarely used (3%). Auscultatory wheezing was detected in 12% of children, while 6% had audible crackles.

### Clinical consequences of chest X-rays

Only three cases (1%) prompted further diagnostic steps due to abnormal CXR results. In one case, a tuberculin skin test was performed after the detection of an infiltrate, but the result was negative. In two other cases, an enlarged cardiac silhouette led to echocardiography, which revealed no abnormalities. No CXR result led to changes in either therapeutic management or the initial diagnosis.

### Spectrum of radiographic findings

CXRs were performed on the day of presentation in 35% of cases, while the majority (52%) were conducted prior to the appointment (Table 2). Despite all children being asymptomatic at the time of imaging, abnormal findings were reported in 73% of cases. These abnormalities were classified into distinct categories, with peribronchial cuffing being the most frequent (35%), followed by peribronchial opacities (13%), parenchymal consolidation (13%), accentuated bronchial markings (13%), pulmonary hypertransparency (11%), and other findings (2%). Pathologic findings were occasionally reported in combination, such as peribronchial cuffing with hypertransparency.

**Table 2:** Chest X-ray findings and its clinical implications. Values are presented as counts (n) with percentages (%). Percentages are calculated relative to the total number of cases (*n* = 271)

Category	Parameter	*n* (%)
Timing of X-ray relative to the clinical visit	Prior	139 (52%)
	Same day	94 (35%)
	Subsequent	38 (14%)
Final assessment of X-ray	pathologic	197 (73%)
	normal	74 (27%)
Pathologic findings	Peribronchial cuffing	95 (35%)
	Peribronchial opacities	36 (13%)
	Parenchymal consolidation	36 (13%)
	Accentuated bronchial markings	36 (13%)
	Pulmonary hypertransparency	30 (11%)
	Other	6 (2%)
Clinical implications of Chest X-ray	Further diagnostics initiated	3 (1%)
	Change in therapy	0 (0%)
	Change of diagnosis	0 (0%)

### Clinical characteristics associated with abnormal chest X-ray findings

Neither sex nor age were significant predictors of abnormal CXR findings. While children aged 4–5 years exhibited the highest proportion of abnormal findings (88%) and those aged 1–2 and 3–4 years the lowest (66% in both groups), these differences were not statistically significant. Premature birth did not increase the likelihood of abnormal findings. The timing of wheezing onset and type (viral vs. multiple-trigger) did not affect CXR results. Similarly, neither personal nor parental history of atopic diseases (allergic rhinitis, atopic dermatitis, or food allergies) increased the risk of pathologic findings. Children with elevated eosinophil counts (≥ 150/µL) had significantly more abnormal CXRs compared to those with lower counts (92% vs. 71%, χ²=5.18, OR 4.43; 95% CI 1.15–17.09, *p* = 0.023). However, total IgE levels and sensitizations to food or aeroallergens were not associated with X-ray outcomes.

There was no significant difference in the proportion of pathologic findings between children treated with inhaled corticosteroids and those who were not. Among children with elevated eosinophil counts (≥ 150/µL, *n* = 47), approximately half received inhaled corticosteroids (51%, *n* = 24). In this subgroup, the percentage of abnormal CXR findings was not significantly different between those who received corticosteroids (96%, *n* = 23) and those who did not (87%, *n* = 20). Albuterol and montelukast treatment did not influence X-ray outcomes. All children using long-acting beta₂-agonists had abnormal CXRs, but statistical significance was not achieved.

In cases where CXRs were performed on the day of presentation to the outpatient clinic, wheezing detected on auscultation had no significant effect on the X-ray findings. All children with audible crackles had abnormal CXRs; however, this association was not statistically significant.

## Discussion

### Cohort characteristics

The involvement of community pediatricians and tertiary care specialists enhanced diagnostic accuracy and consistency. Most children were male (63%), consistent with reports of a higher prevalence of preschool wheezing in boys [12]. Episodic viral wheezing was the most frequent phenotype (90%), though this may be overestimated due to clinical classification; diary-based methods often yield lower rates [13]. Atopic dermatitis affected 10% of children, and among those tested for specific IgE, 31% showed sensitization to aeroallergens. These rates match the general prevalence of atopic dermatitis and aeroallergen sensitization in this age group in Germany [14]. Thus, the study population was not markedly atopic. Considering the male predominance, the high rate of episodic viral wheezing, and the prevalence of atopic diseases and sensitizations, the analyzed cohort reflects a real-world scenario for children with recurrent wheezing in Germany. The study findings may also be applicable to other Western countries with similar healthcare systems and environmental factors. Elevated blood eosinophil counts were the only clinical characteristic associated with abnormal CXR findings, suggesting possible eosinophilic airway inflammation in a subset of children. Nevertheless, this association did not translate into a clinically useful strategy for selecting children who might benefit from chest radiography, as no demographic or clinical characteristics consistently identified a subgroup in whom imaging influenced subsequent diagnostic evaluation or management.

### Clinical consequences of chest X-rays

The principal finding of this study was that chest radiographs rarely influenced diagnostic evaluation or clinical management in preschool children with recurrent wheezing, despite abnormal radiographic findings being reported in nearly three-quarters of cases. This discrepancy indicates that most abnormalities represented nonspecific airway changes rather than findings requiring additional investigations or therapeutic modifications. Only three cases (1%) warranted further evaluation, none of which resulted in new diagnoses or changes in management. These findings indicate that no clinically useful combination of history, physical examination, or laboratory findings emerged that reliably identified children in whom CXR altered subsequent diagnostic evaluation or management.

Our findings are cosistent with current clinical guidelines on recurrent wheezing, which do not consider CXRs an essential diagnostic tool, nor do they base therapeutic decisions on CXR findings [3, 5, 7]. For instance, the 2016 American Thoracic Society (ATS) guideline on *Diagnostic Evaluation of Infants with Recurrent or Persistent Wheezing* reviewed multiple diagnostic options but did not explicitly address CXRs, considering them “generally standard of care” [7]. The most recent European Respiratory Society (ERS) statement on preschool wheezing does not discuss CXRs at all [3], whereas the previous ERS guideline on wheezing disorders in preschool children explicitly states that chest radiographs do not aid in diagnosis or treatment in this population. This conclusion was based primarily on a study by Hederos et al., and recommends imaging only in severe or atypical cases [5, 15]. In summary, existing recommendations have been largely derived from expert opinion and extrapolated from studies involving children with bronchial asthma. The present study provides real-world evidence supporting these recommendations in a well-defined cohort of preschool children with recurrent wheezing.

The minimal clinical impact of CXR findings in our study may partly reflect the study population, which consisted of children with recurrent wheezing in the absence of known chronic pulmonary or cardiac disorders. In contrast, children presenting with persistent respiratory symptoms, such as chronic cough, persistent wheezing between episodes, recurrent lobar disease, or suspected congenital abnormalities, represent a different clinical population in whom chest radiography remains an important diagnostic tool. Certain fixed airway abnormalities may also be suspected on chest radiography. In our tertiary pediatric pulmonology practice, however, structural airway disorders are typically suspected on the basis of such atypical or persistent clinical features rather than episodic wheezing alone and generally require dedicated imaging or bronchoscopy for definitive diagnosis. Furthermore, the static nature of chest radiography limits its ability to assess dynamic airway abnormalities, such as bronchomalacia, which are more effectively evaluated by bronchoscopy.

Taken together, these findings suggest that, although chest radiographs frequently demonstrate nonspecific airway abnormalities, they rarely alter immediate clinical decision-making. Thus, our findings are consistent with current recommendations advocating a clinically driven rahter than routine use of chest radiography in preschool children with recurrent wheezing.

### Spectrum of radiographic findings

Most CXRs were pre-scheduled by the outpatient clinic, allowing for timely discussion during the consultation. Remarkably, three-quarters of the radiologic reports were classified as pathologic. The most common finding, observed in about one-third of the reports, was peribronchial cuffing. It is characterized by thickened bronchial walls and serves as a marker of lower airway inflammation and obstruction [16]. Additionally, some reports described accentuated bronchial markings, which likely represent similar airway changes to those seen with peribronchial cuffing but of lesser extent. Pulmonary hypertransparency was another frequently observed feature and represents the radiographic correlate of pulmonary hyperinflation, often accompanying signs of airway obstruction. Peribronchial opacities and parenchymal consolidation were less common and indicative of more extensive inflammation affecting the lung parenchyma. In two cases, a parenchymal consolidation was interpreted as atelectasis. Other findings were rarely documented. No radiographic findings suggested a specific underlying structural cause of recurrent wheezing. Instead, the observed abnormalities were predominantly nonspecific and consistent with inflammatory airway disease in early childhood. Although parenchymal involvement was occasionally present, the overall radiographic pattern was dominated by airway-related abnormalities typical of preschool children with recurrent wheezing.

### Clinical characteristics associated with abnormal chest X-ray findings

No demographic or anamnestic factorswere associated with abnormal CXR findings. Elevated blood eosinophil counts were the only clinical characteristic associated with abnormal CXR findings (92% vs. 71%, *p* = 0.023). Although these radiographic abnormalities rarely resulted in changes in management, they suggest that lower airway inflammation may persist even outside acute respiratory episodes. Possible explanations include clinically silent viral infections or prolonged post-infectious inflammation. Indeed, bronchoalveolar lavage studies have demonstrated asymptomatic rhinovirus infection in approximately one-third of preschool children with severe recurrent wheezing [17], while sputum analyses have shown increased neutrophilic and eosinophilic airway inflammation in this population [18].

In bronchial asthma, blood eosinophil levels are moderately associated with bronchial eosinophilic inflammation [19]. Similarly, elevated blood eosinophil counts in our cohort may reflect eosinophilic airway inflammation, which could contribute to the observed radiographic abnormalities. However, in preschool children with recurrent wheezing, this relationship remains poorly defined, with no consensus on pathophysiological mechanisms or blood eosinophil thresholds [20–23]. Emerging evidence suggests that blood eosinophilia in preschool children predicts a higher risk of later asthma development and might warrant treatment with ICS [3]. Moreover, ICS are recommended on a trial basis in certain guidelines [5, 24]. In this study, approximately half of the children with elevated eosinophil counts were treated with ICS, yet radiologic outcomes did not differ. Similarly, no difference was observed between children treated with ICS and those untreated, regardless of eosinophilic inflammation. Despite this association, elevated blood eosinophil counts did not identify a subgroup of children in whom chest radiography resulted in clinically meaningful diagnostic or therapeutic consequences.

### Side effects of chest X-rays

Before undertaking any diagnostic procedure, it is essential to weigh its risks against its benefits. The side effects of CXRs include exposure to ionizing radiation, with an estimated effective dose of approximately 0.02 mSv [25]. Although this dose is very low, minimizing unnecessary radiation exposure remains a priority—particularly in children, who are more sensitive to radiation than adults and, due to their longer life expectancy, face a higher cumulative risk of developing malignancies with latency periods spanning several decades [26]. Additionally, the procedure can be a source of stress for both the child and their parents. Young children, who often need to be physically restrained to prevent movement during imaging, may experience fear, anxiety, crying, tachycardia, or elevated blood pressure as a result. In certain instances, such distressing experiences may have long-term psychological effects, including fear or post-traumatic stress [27]. These negative associations with medical environments can persist and may adversely impact the child’s willingness to undergo future medical or dental procedures, potentially influencing their engagement with healthcare into adulthood. Lastly, and although not strictly a side effect, CXR incurs financial costs, which vary by region and healthcare system.

### Strengths and limitations of this study

This study has several limitations. As a single-centre, retrospective analysis, selection bias cannot be excluded, and the findings may not be fully generalisable to other healthcare settings. Because of the retrospective design, variables reflecting disease severity, such as wheezing control, number of wheezing episodes, and symptom duration before presentation, were not documented in a sufficiently standardized manner to allow reliable analysis. As a tertiary pediatric pulmonology outpatient clinic, our study population may have included children with more severe recurrent wheezing than those managed exclusively in primary care. Children with pre-existing pulmonary or cardiac disease were excluded to avoid confounding, which may limit broader applicability. Because rare structural disorders requiring specific treatment were not encountered in this cohort, the present study cannot exclude that chest radiography may provide clinically important information in selected children with atypical presentations. Race and ethnicity data were inconsistently recorded and thus could not be analyzed. This limits the ability to explore potential disparities in radiologic findings or clinical outcomes across different population groups. Incomplete laboratory data also limited subgroup analyses.

Nonetheless, the study includes a relatively large cohort, enhancing statistical reliability. The use of real-world clinical data and systematic characterization of a clinically relevant group—preschool children with recurrent wheezing—strengthens the study’s external validity. By combining the assessment of the clinical consequences of chest radiography with a detailed characterization of radiographic findings and associated clinical characteristics, this study provides a comprehensive real-world evaluation of the diagnostic utility of chest radiography in preschool children with recurrent wheezing.

## Conclusion

This study provides valuable insights into CXR findings in preschool children with recurrent wheezing. Despite a high prevalence of radiologic abnormalities, the clinical consequences were minimal. Elevated blood eosinophil counts were associated with increased rates of radiologic abnormalities, suggesting possible eosinophilic airway inflammation in a subset of children. However, no consistent demographic or clinical predictors emerged. Notably, radiographic signs consistent with lower airway inflammation were observed even outside acute episodes, suggesting that airway inflammation may persist beyond symptomatic periods in a subset of children. Considering the risks of unnecessary radiation exposure and the emotional burden of imaging procedures, these findings are consistent with current recommendations advocating a clinically driven rather than routine use of chest radiography. A comprehensive clinical assessment should remain the cornerstone of diagnosis and management in preschool children with recurrent wheezing.

## Data Availability

The data that support the findings of this study are available from the corresponding author upon reasonable request.

## Abbreviations

BDL: Below detection limit
CXR: Chest X-ray
ICS: Inhaled corticosteroids
IgE: Immunoglobulin E
mo: Months
OR: Odds ratio
RR: Relative risk
SD: Standard deviation
SPSS: Statistical Package for the Social Sciences
wks: Weeks
yrs: Years
